# Leptospirosis in humans and animals in Malaysia: A review from 1976 to 2023

**DOI:** 10.14202/vetworld.2025.673-685

**Published:** 2025-03-23

**Authors:** Joy Siang Xin Lea, Mohd Farhan Hanif Reduan, Siew Shean Choong, Intan Noor Aina Kamaruzaman, Peck Toung Ooi, Sazaly AbuBakar, Shih Keng Loong, Mohammad Sabri Abdul Rahman

**Affiliations:** 1 Public Health and Zoonotic Diseases Research Group, Faculty of Veterinary Medicine, Universiti of Malaysia Kelantan, Pengkalan Chepa 16100, Kelantan, Malaysia; 2 Department of Veterinary Clinical Studies, Faculty of Veterinary Medicine, Universiti Putra Malaysia, Serdang 43400, Selangor, Malaysia; 3 Tropical Infectious Diseases Research and Education Centre, Higher Institution Center of Excellence, Universiti Malaya, 50603, Kuala Lumpur, Malaysia

**Keywords:** leptospirosis, epidemiology, outbreak, zoonosis, diagnostic methods, One Health

## Abstract

Leptospirosis is a globally distributed zoonotic disease that remains under-reported and misdiagnosed, particularly in tropical regions such as Malaysia. This review provides a comprehensive analysis of leptospirosis cases in humans and animals in Malaysia from 1976 to 2023, examining trends in prevalence, outbreak patterns, diagnostic advancements, and associated risk factors. The disease is primarily transmitted through direct contact with infected animals or indirectly via contaminated water and soil, with rodents serving as a major reservoir. In Malaysia, leptospirosis prevalence has increased in recent years, with a notable correlation between outbreaks and occupational exposure, recreational water activities, and monsoon-related flooding. Surveillance data indicate that specific populations, including agricultural workers, town service employees, and animal handlers, are at elevated risk. Furthermore, the disease is commonly misdiagnosed due to its clinical similarities with other endemic febrile illnesses, such as dengue fever and malaria. Advances in diagnostic methodologies, particularly the increasing use of molecular techniques such as polymerase chain reaction (PCR), have enhanced early detection, although serological tests remain widely used in epidemiological studies. This review underscores the necessity of a One Health approach, integrating human, animal, and environmental health strategies to improve surveillance and control measures. Future research should focus on strengthening diagnostic capabilities, understanding environmental reservoirs, and implementing targeted public health interventions to mitigate leptospirosis transmission in Malaysia.

## INTRODUCTION

**Figure 1:**
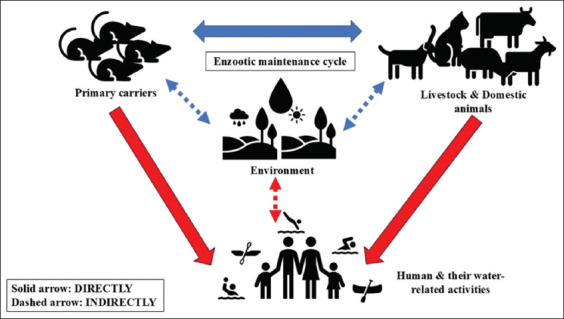
Transmission pathway of leptospirosis infection [3].

Leptospirosis is a zoonotic disease caused by pathogenic spirochetes from the genus *Leptospira*. The bacteria are globally distributed, with an estimated 1.03 million cases annually [1]. This disease is endemic, particularly in poor tropical or subtropical countries, because of its warm and humid climates, and thus provides an optimal breeding ground for the bacteria to survive. It is acknowledged by the World Health Organization that leptospirosis is a neglected disease that is often either misdiagnosed or under-reported [2, 3]. This disease manifests as nonspecific clinical signs in the initial acute phase. However, it can quickly turn into a fatal disease with multiorgan failure when the immune phase starts, and hence, it poses a significant public health concern. Case fatality has been reported to reach up to 6.85% [4]. Leptospirosis can be classified serologically, with approximately 300 serovars that can be grouped into several serogroups [5]. Another classification system based on DNA reassociation resulted in 14 named species, including pathogenic leptospires, non-pathogenic saprophytes, and intermediate pathogenic leptospires [6]. The disease is transmitted directly or indirectly, as illustrated in Figure 1 [3]. This particular disease is carried by various hosts, wildlife, domestic animals, and livestock, all of which have equal opportunities to reservoir the bacteria. In animals, leptospirosis manifests differently. Livestock with the disease usually have reproductive difficulties. In contrast, domestic animals will have similar clinical signs as humans, such as jaundice and renal and/or liver disease [7].

Leptospirosis in animals is an important issue, as it can have a significant economic impact on the country. Economic losses usually occur due to chronic infections that disrupt normal reproductive function in livestock, causing poor performance by the animals [8–10]. Leptospirosis is endemic in Malaysia, with more cases reported in recent years. Several outbreaks with high fatalities have occurred and have been associated with the monsoon, which has led to heavy rainfall and flooding. An outbreak occurred following a search and rescue operation in Lubuk Yu, Pahang, where 153 people were exposed in it [11]. Heavy rain during the first 2 days of the operation caused a disturbance in the soil bed near the river, which was believed to expose and release the pathogen into the water. Unfortunately, three out of four patients who were involved in the study passed away from the infection. Despite that, it was said that leptospirosis is still under-reported [12]. Even though the disease is slowly catching the attention of the public with its rising incidence, we have yet to properly understand the disease despite extensive studies.

Despite the increasing incidence of leptospirosis in Malaysia, the disease remains under-reported and often misdiagnosed due to its non-specific clinical presentation and similarities with other febrile illnesses. While various studies have explored leptospirosis in both humans and animals, there is limited comprehensive analysis of its long-term epidemiological trends, predominant serovars, and the effectiveness of different diagnostic approaches in Malaysia. In addition, the role of environmental reservoirs and cross-species transmission remains underexplored, highlighting the need for a more integrated approach to surveillance and control.

This review aims to provide a comprehensive analysis of leptospirosis in Malaysia by examining its prevalence, outbreak patterns, risk factors, and diagnostic methods in both human and animal populations from 1976 to 2023. It highlights key epidemiological trends and identifies gaps in the current knowledge to support future research and public health interventions.

## LEPTOSPIROSIS IN HUMANS IN MALAYSIA

### Prevalence trends and population involved in human leptospirosis

Reports or studies on human leptospirosis in Malaysia date all the way back to 1925 when the first case of fatal human leptospirosis was diagnosed [13]. Onward, more investigations and surveillance were conducted, and it was revealed that a high prevalence of leptospirosis infection was indeed circulating in humans, and more often than not, it was misdiagnosed due to its similar manifestations of clinical symptoms to other diseases, especially in febrile illnesses. Table 1 [14–27] lists the summary of 14 papers looking at the prevalence of leptospirosis in humans in Malaysia. From 1976 to 1984, Brown *et al*. [14, 15] investigated the cause of febrile illness in patients in Negeri Sembilan and Pahang. The prevalences reported in these studies were not significantly different. It is interesting to note that Brown *et al*. [15] reported leptospirosis among other causes of febrile illness, such as scrub typhus, typhoid/paratyphoid, flavivirus infection, and malaria, which all share similar clinical symptoms. This could lead to a misdiagnosis of the illness and under-reporting of the disease, which could possibly cause a huge gap of empty years without research papers on leptospirosis between 1984 and 2012. More investigations were carried out after leptospirosis was denoted as a notifiable disease in 2010. Most papers involve populations of workers with a greater risk of exposure to leptospirosis, such as agricultural workers, sanitarian workers, farmers, or animal caretakers [16–19, 22–27]. Town service workers or sanitarian workers expose themselves to risk by working all day long in close contact with garbage waste, sewers, drains, market areas, or any environment that is possibly contaminated by rodents when they shed the bacteria through urination [16, 19, 24, 25]. The prevalence of leptospirosis among town service workers increased from 2012 to 2017. However, a different scenario was observed in Sabah in 2020, with the same population of town service workers and urban sanitary workers. 9.4% of the workers were found positive by Atil *et al*. [24], but a high 43.8% seropositivity was reported by Jeffrey *et al*. [25]. The stark difference in prevalence between these studies in Kelantan and Sabah can be attributed to the different diagnostic methods used. Atil *et al*. [24] used polymerase chain reaction (PCR) in their study, which yielded a low result. Jeffrey *et al*. [25], on the other hand, used a microscopic agglutination test (MAT), but they had set the titer to be 1:50 as diagnostic of leptospirosis, which is lower than what was suggested as the cut-off point titer for MAT, 1:100, which leads to the higher prevalence reported. In addition to service workers, animal handlers are also at risk of exposure to the disease.

**Table 1:** Prevalences of leptospirosis in the human population and their type of occupation.

Year	Location	Population involved	Prevalence (%)	Diagnostic method	References
1976	Negeri Sembilan Pahang (Bukit Mendi and Mentakab)	Febrile patients	6.0 (41/688) 4.4 (19/431) and 8.2 (26/318) Overall: 6.0	Hemolytic test (4-fold rise in titer)	[14]
1984	Pahang	Febrile patients from urban, rural, and oil palm workers.	6.8 (110/1629)	Hemolytic test (≥1:320)	[15]
2012	Kelantan	Town service workers - Garbage collector - Town cleaner - Landscaper - Lorry driver	24.6 (overall) 20/296 27/296 19/296 7/296	MAT	[16]
2013	Northeastern Malaysia	Febrile patients - Agriculture workers - Military - Medical personnel - Outdoor workers	8.4 (overall) 29/84 4/84 2/84 12/84	IgM ELISA, MAT	[17]
2016	Melaka Johor	Oil palm plantation worker	28.6	MAT	[18]
2017	Kelantan	Town service workers 2@- Garbage collector - Town cleaner - Lorry driver/mechanics - Landscaper	25.5 (overall) 13/321 47/321 14/321 8/321	MAT	[19]
2018	Selangor	Wet market workers Food handler	22.5 (52/231) 23.8 (55/231)	MAT, IgM, and IgG ELISA	[20]
2018	Kelantan	Wet market workers	33.6 (78/232)	MAT	[21]
2018	Northeastern Malaysia	Cattle farmers	72.5	MAT	[22]
2019	Perak	Rangers	40 (2/5)	MAT	[23]
2020	Sabah	Town service workers - Garbage collector - Urban sweeper - Lorry driver - Landscaper	9.4 (overall) 2.3 (9/394) 3.8 (15/394) 1.3 (5/394) 2.0 (8/394)	PCR	[24]
2020	Sabah	Urban sanitary workers	43.8 (133/303)	MAT	[25]
2020	Johor Kuala Lumpur (KL) N. Sembilan Selangor	Shelter dog handlers Working dog handlers	23.7 (46/194) 10.8 (21/194)	MAT	[26]
2020	Southern Malaysia	Dog and cat caretakers in animal shelters	8.62 (5/58)	MAT	[27]

MAT=Microscopic agglutination test, PCR=Polymerase chain reaction, ELISA=Enzyme-linked immunosorbent assay, IgM=Immunoglobulin M, IgG=Immunoglobulin G

A high seroprevalence of 72.5% was reported in cattle farmers in Kelantan. The predominant serovar was a local strain known as Sarawak, where the pathogenicity information of the strain was minimal, but some studies detected the presence of the Sarawak strain in wildlife like squirrels and bats [25]. Animal caretakers such as dog handlers, cat caretakers, and rangers work in close proximity with their counterparts, which facilitate the transmission of the pathogens easily among themselves. 8.62%–23.7% seropositivity was observed in cats and dogs caretakers [26, 27]. The shelters were located in rural areas surrounded by forest or oil palm plantations, which allowed the encroachment of rodents or small mammals into the shelter environment, which may cause enzootic transmission between the animals. Hygiene practices around shelters, animal density of shelters, and personal protective equipment (PPE) are factors that must be considered in looking into the risk factors of infection in humans and animals [26, 27]. Hence, leptospirosis is considered an occupational disease or hazard and is one of the risk factors for the disease.

## OUTBREAK CASES IN MALAYSIA

Besides being an occupational disease, as described previously, leptospirosis is also closely related to recreational activities involving water, especially after a flooding period. In this review, four outbreaks of leptospirosis are discussed. First, an outbreak of leptospirosis was declared in October 1999 in Sabah [28]. Forty six males, aged 8–19 years old, with a history of swimming in the creek, were admitted to Beaufort Hospital, Sabah; 30 were presented with fever, vomiting, body aches, giddiness, headache, chest pain, and cough. A case-control investigation was performed. The MAT result of paired sera showed 18 positive results at titer ≥360 with 8 paired sera indicating current infection. Unfortunately, one patient passed away from hemorrhagic shock due to pulmonary hemorrhage and a clinical diagnosis of leptospirosis was made. Heavy rainfall during the 1^st^ week of October caused flooding in an area near the creek that was surrounded by agricultural activity with commercial livestock farming. Flooding may have caused the spread of organisms from the soil into the creek, exposing the risk to the animals that swim in the creek. Since leptospirosis was not mandated as a notifiable disease in 1999, the symptoms can easily be under-reported or misdiagnosed as another disease. Medical officers must be alert and consider leptospirosis as a differential diagnosis to provide early treatment to patients. The public should also be aware of the potential risk of swimming in a creek in a possible endemic area. The second outbreak occurred in 2000, during a multisport event, “Eco-Challenge” in Sabah, involving international athletes [29]. The Centers for Disease Control was notified when there was a surge of febrile illness cases with an acute onset of high fever, chills, headache, and myalgias involving all patients that had participated in the “Eco-Challenge.” The epidemiologic investigation was performed to identify the cause of the illness. Participants were traced back and interviewed about the details of the race activities, geography of the course, and possible exposures. Serum samples were sent for immunoglobulin M (IgM) enzyme-linked immunosorbent assay (ELISA) testing and confirmed with MAT with titer ≥200 was considered positive. Samples were also tested against alternative organisms such as dengue, rickettsia, Japanese encephalitis, Chikungunya, Snowshoe hare virus, and hantavirus. 26/38 serum samples sent were tested positive with the IgM ELISA test and 20 were subsequently confirmed positive with MAT. Serovar Australis was found to react strongly with the samples. No deaths were reported during this outbreak. Results from the investigation revealed that doxycycline may have a protective effect and can be used as a prophylaxis against leptospirosis before traveling to an endemic country. The infection attack rate was lower in athletes who took doxycycline during the race period. Multiple water-related activities during the race, including prolonged swimming in the river, kayaking, spelunking, and accidentally swallowing river water, were considered significant risk factors for the illness, according to the univariate analysis. However, the multivariate stepwise logistic regression showed that only swimming was independently associated with the illness. Once again, heavy rainfall, which happened before the race, may have caused the stagnation of water in the area, saturating the soil with leptospires. Athletes participating in the event had to do activities such as jungle trekking and going into the mud near the riverbank, exposing themselves to the risk of infection. Traveling to other countries needs to be planned well. Protect yourself with information about the country and its endemic diseases. Prepare PPE or heavy-duty clothing if traveling into the forest or engaging in water-related activities. In 2010, after a search and rescue operation in Lubuk Yu, Pahang, 153 people were believed to have been exposed to leptospirosis. Four tested positive for leptospirosis co-infecting with melioidosis and had diabetes mellitus, hypertension, or heart disease. Three patients died, resulting in a high case fatality rate of 75%. Garbage and food waste were accumulated around the recreational area during rescue operation days, which attracted rodents to the area and caused river contamination [29, 30]. A more controlled and well-planned search and rescue operation, led by professionals using appropriate PPE, should be implemented. Rescuers and volunteers need to be screened for any chronic diseases before joining the operation, and follow-up on the health status of all related personnel after the operation is a must. Clean drinking water should also be available to the team. Direct drinking of river water is highly not advised, especially in search and rescue operations. Lastly, this outbreak occurred in 2016 when Neela *et al*. [31] conducted an epidemiologic investigation among the reserve military recruits after a survival exercise in Hulu Perdik Forest. Twelve military recruits presented with high-grade fever, chills, headache, myalgia, diarrhea, and vomiting. Blood samples were analyzed using Lepto IgM rapid, IgM ELISA, and MAT. Environmental samples such as soil and water were also included in the test. Rodents were caught, and their kidneys were harvested for culture and isolation. Neela discovered from her investigation that all recruits had taken prophylactic antibiotic treatment before the training, and 91.6% of them accidentally swallowed river water while swimming. Two of them were positive in MAT serovar in response to Autumnalis and Hardjobovis. 6 rodents, 6 water, and 8 soil samples were positive under dark field microscope examination. All samples were positive for pathogenic *Leptospira* through PCR. Phylogenetic analysis of the environment and animal samples confirmed the presence of *Leptospira interrogans* and *Leptospira borgpetersenii* in the rodents; *Leptospira kmetyi* and *Leptospira wolffi* were found in the soil and water samples. No deaths were reported during this outbreak. It is interesting to see that neither rainfall nor flooding happened in this outbreak, but pure contamination of the environment through the presence of carrier animals (rodents) is enough to cause infection in humans if they are not being careful. The recruits were not aware of the potential risk factors of leptospirosis. No boots were worn, and no protective clothing was put on. Pre-exposure prophylaxis provided a protective effect similar to what happened in the Eco-Challenge. Awareness of the potential risk factors of the disease should be given to high-risk populations. PPE is always essential in water-related activities. This outbreak showed an intricate epidemiological relationship of leptospirosis through the presence of rodents within the establishment environment with the human population within the proximity of the place contaminated the soil and water.

## CASE REPORTS OF HUMAN LEPTOSPIROSIS IN MALAYSIA

Eleven leptospirosis cases reported from 2007 to 2022 were included in this review (Table 2) [14, 29, 32–40]. The majority of the cases were co-infections with other febrile illnesses that are endemic in Malaysia. Most initial clinical symptoms include fever, myalgia, arthralgia, diarrhea, or vomiting. Fatality increases in patients with co-infection. Two separate interesting, unexpected cases with a rare presentation of neurological symptoms and ichthyosis were reported, which were considered uncommon and rarely recognized in leptospirosis patients [32, 38]. No association has been reported between ichthyosis and leptospirosis, and skin breakage was believed to have encouraged the entry of pathogens [32]. Most of the diagnostic methods used were a combination of serological analysis, such as *Leptospira* IgM or immunoglobulin G ELISA, a rapid test kit that was usually confirmed with the gold standard diagnostic test, MAT. A few studies [30, 32, 35, 37, 39] also indicated the use of a molecular diagnostic method, namely PCR.

**Table 2:** Summary of case reports on leptospirosis in humans in Malaysia from 2007 to 2022.

Disease details	Initial clinical symptoms	Diagnostic methods	Complications	References
Leptospirosis with acute large-bowel gangrene	Fever, myalgia, diarrhea, and jaundice	Positive *leptospira*l IgM and IgG levels	ARDS, acute renal failure, bowel perforation (Alive)	[14]
Coinfections of leptospirosis and melioidosis	Fever, myalgia, arthralgia, diarrhea, and vomiting	PCR	Case 1: Right knee septic arthritis, hypotension (Dead) Case 2: ARDS (Dead) Case 3: ARDS, acute renal failure, hypotension, atrial fibrillation (Case 4: Unstable angina (Alive)	[29]
Leptospirosis with acquired ichthyosis	Fever, dry, itchy thickened skin	MAT, PCR	None (Alive)	[32]
Malaria and leptospirosis coinfections	Fever, rigor, headache, myalgia, fatigue, and anorexia	Leptospirosis IgM dipstick, MAT	None. (Alive)	[33]
*Leptospira*l infective endocarditis concurrent with dengue	Fever, arthralgia, myalgia, nausea, vomiting, lethargy	*Leptospira* IgM ELISA, MAT	Severe type I respiratory failure, severe mitral regurgitation, and tricuspid regurgitation with vegetation (Alive)	[34]
Coinfections of melioidosis and leptospirosis	Fever, chills, and rigor; headache; arthralgia; myalgia; epigastric pain	PCR	Respiratory distress, septic shock (Dead)	[35]
Coinfection of dengue and leptospirosis	Fever, generalized bodach hemoptysis	*Leptospira* IgM ELISA, MAT	Fulminant pulmonary hemorrhage (Dead)	[36]
Subclinical leptospirosis in pregnant women	Bilateral thigh pain and fever	Serological analysis revealed positivity for *Leptospira* IgM by PCR	Brought unconscious to the emergency department, died shortly after arrival	[37]
Neuroleptospirosis	Jerky movements of the whole body, saliva drooling, flu-like illness, fever, myalgia, headache	*Leptospira* IgM detected in the MAT	Anton’s syndrome, no follow-up, patient went back to China (Unknown)	[38]
Leptospirosis	Fever, mild flu-like symptoms, headache, myalgia, diarrhea, and vomiting	PCR	Dead upon arrival. (Dead)	[39]
Coinfections of melioidosis and leptospirosis	Fever, abdominal pain, and jaundice	MAT	Septic shock complicated by multiorgan failure (Dead)	[40]

MAT=Microscopic agglutination test, PCR=Polymerase chain reaction, ELISA=Enzyme-linked immunosorbent assay, IgM=Immunoglobulin M, IgG=Immunoglobulin G

Interestingly, leptospirosis infections in different living settings, such as rural versus urban settings, show a large distinct difference in prevalence, as shown by Suut *et al*. [41] and Sahimin *et al*. [42], respectively. A higher prevalence of leptospirosis was detected in rural areas (37.4%) than in urban settings (12.4%). Even though the differences are quite large, the affected populations are always due to almost similar risk factors, such as the presence of water bodies.

## LEPTOSPIROSIS IN ANIMALS IN MALAYSIA

### Role of reservoirs, prevalences, and predominant serovars

Studies on the prevalence of leptospirosis in animals have been quite extensive over the years, especially in recent years. More papers were published after the 2000s. The earliest papers date back to 1987 and 1988, when a cross-sectional serological survey of leptospiral infection in domestic animals was conducted in West Malaysia by Bahaman *et al*. [43, 44]. Livestock such as cattle, buffalo, goats, sheep, and pigs were also included in the study. Results showed quite a high prevalence of disease in cattle, buffaloes, and pigs, accounting for 40.5%, 31.0%, and 16%, respectively [43]. The changes in the prevalence of each livestock are listed in Table 3 [43–48]. The prevalence of this disease increased over time for cattle, buffalo, goats, and sheep. Compared with cattle and buffalo, goats, and sheep had a lower prevalence of the disease. The serovars that were majorly associated with the cattle and buffaloes were the serovars Hardjo and Pomona in pigs and sheep. Interestingly, the serovar Sarawak was found to be predominant in cattle, as reported by Daud *et al*. [45]. Serovar Sarawak is a local strain that was commonly not included in a routine panel of live antigens for leptospirosis MAT; hence, it was rare to detect in other studies, and the high seropositivity was detected in cattle. Pigs, on the other hand, showed a significant decrease in prevalence from 1987 to 2017. This decrease was attributed to shifting the swine industry farming system from extensive to intensive indoor farming, which inadvertently helped restrict the exposure to rodents. The different diagnostic methods used in the studies, MAT and PCR, showed a drastic difference in the detection rate of infection. Even though the results are different, their interpretation is important. A low level of prevalence by PCR is sufficient to demonstrate active infection in the animals because it detects antigens in the samples. High seropositivity from MAT indicates that the animal population was previously exposed to the infection and developed antibodies against it.

**Table 3:** Trend in the prevalence of leptospirosis in livestock population.

Species	Year	Location	Prevalence (%)	Diagnostic method	Predominant serovar	References
Cattle	1987	WM	40.5 (558/1378)	MAT	Hardjo	[43]
	1988^a^	WM	45.5 (42/222)	MAT	Hardjo	[44]
	2018	Kelantan	81.7 (343/420)	MAT	Sarawak	[45]
	2019^b^	Kelantan	0.63 (4/635)	PCR	N/A	[46]
	2021	Kelantan	14.16 (145/1024)	MAT	Hardjobovis	[47]
Buffalo	1987	WM	31.0 (78/133)	MAT	Hardjo	[43]
	1988^a^	WM	45.5 (42/222)	MAT	Hardjo	[44]
Goats	1987	WM	4.4 (29/657)	MAT	Pomona	[43]
	2019^b^	Kelantan	0.63 (4/635)	PCR	N/A	[46]
	2021	Kelantan	11.20 (41/366)	MAT	Hebdomadis	[47]
Sheep	1987	WM	0.8 (3/44)	MAT	Pomona	[43]
	2019^b^	Kelantan	0.63 (4/635)	PCR	N/A	[46]
	2021	Kelantan	5.03 (17/338)	MAT	Pomona	[47]
Pigs	1987	WM	16.0 (139/869)	MAT	Pomona	[43]
	2017	Selangor	6 (5/81)	PCR	Pomona	[48]

a The prevalence was for both cattle and buffaloes, but not for each species.

b This is a prevalence for pooled samples of cattle, goats, and sheep. MAT=Microscopic agglutination test, PCR=Polymerase chain reaction

Table 4 [27, 28, 48–56] summarizes the leptospirosis prevalence in the domestic animal population (cats and dogs). The prevalence was within the range of 3%–42.7% in dogs and 14.89%–25.6% in cats. The number of animals sampled, diagnostic methods, health status, vaccination status, location of where the animals were kept, and hygiene status of their location are some of the factors that dictate the variation in the prevalence of domestic animals. Predominant serovars circulating within domestic animals were mainly dictated by Bataviae, Javanica, Ballum, and Icterohaemorrhagiae.

**Table 4:** Trends in leptospirosis prevalence in domestic animals’ population.

Year/location	Species	Prevalence (%)	Titer	Diagnostic methods	Predominant serovars (Top 3)	References
2020/Johor KL N. Sembilan Selangor	Working dogs: Shelter dogs	6.4 (17/266) 19.9 (53/266) Overall: 26.3 (70/266)	1:100	MAT	Icterohaemorrhagiae Ballum Bataviae	[27]
2020/Southern Malaysia	Shelter dogs Cats	20.47 (26/127) 14.89 (7/47)	1:100	MAT	Bataviae, Javanica Ballum	[28]
2017/Selangor	Stray dog Cats	7.3 (11/150) 0		PCR	Canicola Icterohaemorrhagiae	[48]
2016/NA	Shelter dogs	MAT: 3.8 (3/80) PCR: 0	1:80	MAT PCR	Bataviae	[49]
2016/ Selangor	Healthy dogs Dogs with k/d	MAT: 2.6 (1/38) PCR: 0 MAT: 15.8 (3/19) PCR: 5.3 (1/19) Overall: 7.0 (4/57)	1:80	MAT PCR	Canicola Icterohaemorrhagiae	[50]
2017/NA	Working dogs	MAT: 3.1 (3/96) PCR: 0	1:80	MAT PCR	Australis Bataviae Javanica	[51]
2019/NA	Cats	18.18 (20/110)	1:100	MAT	Bataviae Javanica Ballum	[52]
2019/Johor Selangor	Working dogs and shelter dogs	22.2 (59/266)	1:100	MAT	Icterohaemorrhagie Ballum Bataviae	[53]
2020/NA	Cats	MAT: 25.6 (21/82) PCR: 4.9 (urine) PCR: 8.5 (blood) Culture and isolation- 4.9	1:100	MAT PCR Culture and isolation	Bataviae Javanica Ballum	[54]
2021/Selangor	Dogs with kidney or liver disease	MAT: 42.7 (53/124) PCR: 42.7 (54/124)	1:100	MAT, PCR	Bataviae Javanica Icterohaemorrhagiae	[55]
2021/Selangor	Stray dogs	32 (32/100)	1:100	MAT	Javanica Bataviae Icterohaemorrhagiae	[56]

MAT=Microscopic agglutination test, PCR=Polymerase chain reaction

Another portion of the studies on the prevalence of leptospirosis in animals involved the primary reservoir, which was rodents from different areas in Malaysia. Investigation to identify the circulating serovars in the rats’ population in Kuala Lumpur, the city center of the country, revealed a prevalence of 22.8% (13/57 positive cultures isolated from 112 rats’ kidney samples) detected through MAT and was found to react strongly to the serovars of Bataviae, Javanica, Icterohaemorrhagiae, Australis, and Canicola [57]. In the next study by Kamaruzaman *et al*. [58], the rodent population in Kelantan, more specifically in the wet markets area, was targeted to identify the species of *Leptospira* carried by the rodents. Forty rodents made up of 30 rats and 10 shrews were caught, and 38 kidney samples (2 rats’ kidneys were autolyzed) were extracted for PCR using 16S ribosomal RNA (16S rRNA) and *Lip*L32 genes. The result shows that 52% (20/38) of the rodents were positive for the 16S rRNA gene and 39% were positive for *the lipL32 gene*. Phylogenetic analysis of the positive samples identified *L. interrogans and L. borgpetersenii*. In Sarawak, Pui *et al*. [59] aimed to investigate the status of leptospirosis in national service training centers and paddy fields by sampling the rats, soil, and water samples from the sites. 31 rats were captured, and their kidneys and liver samples were inoculated into modified Ellinghausen-McCullough-Johnson-Harris (EMJH) broth and incubated for 3 months. PCR targeting 16SrRNA, *lipL*32, and *rrs* gene were conducted every month. Out of 31 rats, only 1 (3.2%) was found to be positive for intermediate *Leptospira*. Suut *et al*. [60] focused on rodent sampling in areas close to human dwellings in Sarawak was conducted to determine the seroprevalence of leptospirosis. A total of 241 rodents were caught and euthanized. Blood samples collected through cardiac puncture were subjected to MAT. 40.7% (98/241) seropositivity was detected in the rodent population, with serovars Autumnalis, Tarassovi, Bataviae, Hebdomadis, and Celledoni found to be predominant in the population. Azhari *et al*. [61] investigated the *Leptospira* strains circulating in small mammals in sites where previous human leptospirosis cases were reported in Selangor. Study sites were further categorized into urban residential, semi-urban, and recreational forests according to the characteristics of the geographical area. A total of 266 small mammals, including rats, squirrels, and shrews, were trapped and euthanized before kidney samples were extracted. PCR using *lipL32* gene was performed, and a 14.3% positivity rate was found for four different *Leptospira* species (*L. interrogans*, *Leptospira kirschneri*, *L. borgpetersenii*, and *Leptospira weilii*). Benacer *et al*. [62] also conducted a study on an urban rat population in Kuala Lumpur to isolate and detect *Leptospira* spp. using both MAT and PCR. A total of 300 rats were caught and euthanized. Blood from cardiac puncture, liver and kidneys, and urine samples were inoculated into a modified EMJH medium and incubated for 3 months. 6.7% (20/300) of the culture were positive and confirmed with PCR to be pathogenic *Leptospira* spp. using two types of primers targeting 16SrRNA and *secY* genes. The identification of serogroups using MAT revealed the predominant serovars to be Javanica, followed by Bataviae. From these studies, urban rats, rats in wet markets, rats in forests, and urban or semi-urban residential areas are all carriers of certain *Leptospira*.

From the reviewed papers, leptospirosis was found or detected more often in animals and humans in recent years in Malaysia. This could be due to the government’s directive on gazette leptospirosis as a mandatory notifiable disease [63]. Although the disease is endemic in Malaysia, due to the similar presentation of the clinical signs with other tropical diseases, leptospirosis is often misdiagnosed or under-reported. As reviewed in the paper, leptospirosis patients were usually presented to the hospital with initial symptoms of high-grade fever, which could have differential diagnoses of a few other diseases, such as dengue fever or malaria, which are considered the top ten most communicable diseases in Malaysia [64]. This disease, however, has gained more attention after a significant increase in reported outbreaks, which has resulted in fatalities. Annually, 58,900 deaths were estimated due to leptospirosis worldwide [1]. It is a life-threatening disease despite its neglected disease status. Leptospirosis is also known as biphasic illness with a broad spectrum of severity. Approximately 90% of the time, this disease is self-limited, accompanied by non-specific symptoms, such as fever, myalgia, abdominal pain, or gastrointestinal discomforts such as vomiting or diarrhea [6, 65]. Very rarely is death observed in the acute phase of the illness, which indicates that severe leptospirosis is also very rare. However, this infection can progress to a more severe and potentially fatal condition when the immune phase occurs, which involves multiorgan failures, as indicated in all the fatal case reports in this review. The fatality of severe leptospirosis cannot be undermined as it can go as high as 15% and even higher when pulmonary hemorrhage is involved in a case [66]. At the same time, it is important to realize that the underlying disease that patients have before contracting leptospirosis could lead to a more devastating and progressive disease. Leptospirosis infection on a standalone basis can have a huge impact on health, but the risk increases with coinfection, and patients are shown to face complications at a later stage, which leads to death.

The distribution of leptospirosis burden in Malaysia in this review paper is dictated by the population density that was caused by urbanization, such as in city Centers like Kuala Lumpur or Selangor, where humans are more populated. Specific sites were chosen, such as wet markets, recreational forests, oil palm plantations, or military training grounds, because these places were associated with the presence of rodents either due to poor sanitation or places with water activities that can be the conduit of transmission. The study sites in those reviewed papers were also chosen usually because of an outbreak of leptospirosis. Investigations were usually performed around the area to review the status of the disease and people affected, the presence of possible carriers, or environmental contamination, which could be the risk factors of the infection. In this review, leptospirosis is considered an occupational disease. The nature of the job directly facilitates disease transmission. Town service workers deal with garbage most of the time. Where there is garbage, there will be rats. Rats are known as the primary reservoir of *Leptospira* [67, 68]. They are carriers of disease and can shed pathogens through urination, thus contaminating the environment. Similarly, in wet markets, lots of fresh produce is sold, and this attracts rats as well. In addition to rats, other animals can be carriers of the disease and sources of infection. Humans coming into contact with these animals and being always in close proximity inevitably increase the risk of exposure to the disease [69]. This demonstrates the involvement of disease transmission between humans and animals. In this review, cattle farmers, animal caretakers in shelters, dog handlers, and rangers are among the affected populations. Because leptospirosis is a zoonotic disease, close proximity between animals and humans has become a major risk factor for disease transmission. Each species can be a reservoir and source of infection. When exposed to asymptomatic infected animals, precautions must be taken when handling animals. The predominant serovars circulating in dogs and cats were Bataviae, which are similar to the serovars found in rats. This is significant because serovar Bataviae is not included in the leptospirosis vaccine for dogs, and this serovar was carried by rats, which could only mean that dogs contracted the serovar by exposure to the small mammals. The same serovars were found to be circulating in humans, especially in animal caretakers. This demonstrates how endemic it is for serovars to circulate in Malaysia. Although animal hosts play a major role in the transmission of leptospirosis, hygiene is also a probable risk factor for leptospirosis as well. From the reviewed papers, the hygiene of the location where the animals were kept contributes significantly to the prevalence of the disease. Goh *et al*. [53] concluded in their study that overcrowding in a dog shelter has increased manure output. Limited manpower to clean up the situation invites the presence of rats, which have a high possibility of becoming the main reservoir of the disease; hence, transmission occurs through contamination of the environment. Risk factors of hygiene or sanitation issues were well portrayed in the study conducted in urban slums in Brazil, where the issues were further complicated by poor socioeconomic status of the population, which increases the risk of transmission of leptospirosis [70].

Given the difficulties associated with non-specific clinical signs, a good diagnostic test is essential. Various diagnostic methods were used to confirm the diagnosis or conduct prevalence studies in Malaysia. Table 5 [71–75] presents a brief overview of the comparison of these commonly used diagnostic tests for diagnosing acute phase leptospirosis. The MAT is the gold standard for diagnosing a disease despite its limitations in terms of its laborious process and low sensitivity for diagnosing acute infections [76]. Other serological assays, such as ELISA or rapid diagnostic tests, have since been used in combination with MAT as part of routine diagnostic methods, which have been observed in most clinical settings in Malaysia. However, the antibody titer is usually low in the early phase, which makes the diagnosis based on a single serum sample. This is a challenge in the effective diagnosis of leptospirosis. Molecular detection methods like PCR are recommended to overcome this limitation. It can be used on its own or as a complementary test to existing MAT, ELISA, or rapid diagnostic tests, especially during the early phase of the illness. In Malaysia, the use of PCR has increased more frequently in recent years in clinical settings and in prevalence studies. It is more robust and highly sensitive to detecting pathogens in samples. Choosing the right diagnostic method is important for making the right diagnosis and starting appropriate treatment to prevent possible worse outcomes. Mullan *et al*. [73] demonstrated increased sensitivity for diagnosing when PCR was combined with other serological diagnostic methods. The urgency of early diagnosis is essential to ensure that the right treatment is provided to avoid any complications caused by delayed diagnosis.

**Table 5:** Comparison between MAT, ELISA, and PCR for diagnosing acute-phase leptospirosis.

Criteria	MAT (%)	ELISA (%)	PCR (%)	References
Sensitivity	55.3^b^	86.0^b^	52^c^	[71]^a^
	14^a^	84^c^	62^d^	[72]^b^
Specificity	95.7^b^	84.5^b^	79^c^	[73]^c^
	86a	75^c^	100^d^	[74]^d^
Advantages	- Useful for epidemiological purposes - Identification of infecting serovars	- Available in most laboratories - Comparatively cheaper	- Useful in the acute phase of leptospirosis - Time saving	[71–75]
Limitations	- Laborious and time-consuming - Need a skilled technician - Maintenance of live *Leptospira* strains - Cross-reactions can occur between related serovars	- The procedure requires a skilled technician	- Inability to identify circulating serovar in specific geographical areas - May produce false-negative results when leptospire numbers are low	

MAT=Microscopic agglutination test, ELISA=Enzyme-linked immunosorbent assay, PCR=Polymerase chain reaction

## CONCLUSION

This review highlights the epidemiology of leptospirosis in Malaysia over the past decades, emphasizing its prevalence, risk factors, outbreak patterns, and diagnostic advancements. Leptospirosis remains a significant zoonotic disease in Malaysia, with occupational exposure, water-related activities, and environmental contamination playing crucial roles in its transmission. The disease is prevalent among humans, livestock, domestic animals, and rodents, reinforcing the necessity of a One Health approach in surveillance and control efforts. The increasing use of molecular diagnostic methods, such as PCR, has enhanced early detection, although serological methods remain widely used in epidemiological studies. Despite improvements in awareness and reporting, leptospirosis continues to be underdiagnosed due to its non-specific symptoms and similarities with other tropical infections.

A major strength of this review is its comprehensive analysis of leptospirosis trends in Malaysia, integrating human, animal, and environmental perspectives. This review provides a holistic understanding of the disease’s trajectory, risk factors, and diagnostic advancements over nearly five decades by compiling data from multiple sources. However, limitations exist, including the reliance on previously published studies, potential gaps in under-reported cases, and variations in diagnostic methodologies across studies, which may have influenced prevalence estimates. In addition, there is a lack of large-scale, nationwide studies that systematically assess leptospirosis transmission dynamics across different ecological and occupational settings.

Future research should focus on improving surveillance strategies, particularly in rural and flood-prone regions where outbreaks are common. The role of environmental reservoirs, including soil and water contamination, requires further investigation to develop effective intervention measures. Advancing rapid and cost-effective diagnostic tools and developing targeted vaccination strategies would enhance disease control efforts. In addition, interdisciplinary collaboration among healthcare professionals, veterinarians, and environmental scientists is crucial to strengthening One Health-based approaches for leptospirosis prevention and management in Malaysia.

By addressing these gaps, future studies can contribute to a more comprehensive understanding of leptospirosis epidemiology and guide policymakers in implementing more effective public health interventions.

## AUTHORS’ CONTRIBUTIONS

JSXL and MSAR: Conceptualized the study, collected the literature, and drafted the initial version of the manuscript. MFHR, SSC, INAK, PTO, and MSAR: Developed the theoretical framework on the animal aspects of the study, participated in the collection of literature, analyzed the literature, validated the literature, and critically reviewed, edited and revised the manuscript. SA, SKL, and MSAR: Developed the theoretical framework on the human aspects of the study, participated in the collection of literature, analyzed the literature, validated the literature, and critically reviewed, edited and revised the manuscript. MFHR, SSC, INAK, SA, SKL, and MSAR: Supervised the study. All authors have read and approved the final manuscript.
